# Immunomodulatory responses of peripheral blood mononuclear cells from multiple sclerosis patients upon in vitro incubation with the flavonoid luteolin: additive effects of IFN-β

**DOI:** 10.1186/1742-2094-6-28

**Published:** 2009-10-13

**Authors:** Zohara Sternberg, Kailash Chadha, Alicia Lieberman, Allison Drake, David Hojnacki, Bianca Weinstock-Guttman, Frederick Munschauer

**Affiliations:** 1 Department of Neurology, Baird MS Center, Jacobs Neurological Institute, Buffalo, NY, USA; 2 Department of Molecular & Cellular Biology, Roswell Park Cancer Institute, Buffalo, NY, USA

## Abstract

The study is aimed to determine the role of luteolin (3',4',5,7-tetrahydroxyflavone), alone and in combination with human interferon-beta (IFN-β), in modulating the immune response(s) of peripheral blood mononuclear cells (PBMCs) isolated from multiple sclerosis (MS) patients. PBMC proliferation in the presence or absence of these drugs was determined and the production of pro-inflammatory cytokines (IL-1β, TNF-α), and the ratio of cell migration mediator MMP-9, and its inhibitor, TIMP-1 was assessed in the culture supernatants. Luteolin reduced, in a dose-dependent manner, the proliferation of PBMCs, and modulated the levels of IL-1β and TNF-α released by PBMCs in the culture supernatants. Luteolin reduced the MMP-9/TIMP-1 ratio via lowering MMP-9 production. In the majority of cases, luteolin, when combined with IFN-β, had additive effects in modulating cell proliferation, IL-1β, TNF-α, MMP-9 and TIMP-1.

## Background

Flavonoids, are group of polyphenolic compounds, known to have significant anti-tumor, antioxidant and anti-inflammatory activities [1]. Epidemiological studies have shown that high intake of fruit and vegetables, rich in flavonoids, is protective against various forms of cancer [2], cardiovascular diseases [3] and neurodegenerative diseases [4]. Luteolin, 3',4',5,7-tetrahydroxyflavone, an important member of the flavonoid family has shown to exert immunomodulatory effects that may be beneficial in the treatment of neurodegenerative diseases such as multiple sclerosis (MS), which has an underlying T-cell mediated autoimmune pathology [5].

*In vitro *studies show that luteolin inhibits T cell activation [6] and reduces the proliferation of autoreactive T-cells induced by both alpha B-crystallin and the murine encephalitogen proteolipid protein peptide (PLP), candidate autoantigens in MS and experimental autoimmune encephalomyelitis (EAE) respectively [7]. In addition, luteolin blocks myelin basic protein-induced mast cells' stimulation which are capable of activating T-cells [8,9].

Furthermore, luteolin has been shown to reduce induction of proinflammatory cytokine from LPS-stimulated human peripheral blood mononuclear cells (PBMC) [10], LPS-stimulated dendritic cells [11] and IL-1β-activated astrocytes in culture [12]. A recent study shows that inter-peritoneal administration (50 mg/kg) or oral treatment (100 mg/kg) of luteolin suppresses clinical symptoms of EAE and prevents relapse when administered either before or after EAE disease onset [13]. The EAE suppression by luteolin is related in part to its ability to inhibit mast cell activation [8]. The activation of brain mast cells, which are located perivascularly, results in an increase in blood brain barrier permeability [14] and the release of cytokines and chemokines necessary for the migration of activated T-cells into the CNS, thereby facilitating T-cell-mediated inflammatory processes [15].

Luteolin and its glucoside metabolite, luteolin 7-O-glucoside, are potent inhibitor of MMP-9 activity [16], suggesting that luteolin may interfere with the migration of activated immune cells into the CNS via modulation of proteins crucial for this migration [17]. This notion is supported by an *in vivo *study showing that luteolin treatment prevents monocyte migration across the brain endothelium, resulting in reduction of inflammation and axonal damage in the CNS of the EAE mice [13].

The immunomodulatory effects of luteolin are similar to those of its close relative quercetin (3,3',4',5,7-pentahydroxyflavone). However, *in vitro *and *in vivo *studies show that luteolin has enhanced immunomodulatory activities compared to quercetin [10,13,18-20]. Studies of human PBMC treated with 5 μM flavonoids show that this concentration of quercetin is less effective in reducing TNF-α production, than is a similar concentration of luteolin which reduces TNF-α production by more than 50% [10]. Similar results were shown in studies using murine macrophages [18,19]. In addition, luteolin has been shown to reduce MMP-9 activity in A431 tumor cells more effectively than quercetin [20]. These *in vitro *results are further supported by the *in vivo *study showing that IP administration of 50 mg/kg luteolin from day six after disease induction reduces the mean clinical scores of EAE by approximately 25% while IP administration of luteolin is effective by more than 80% [13].

In a recent study [21], we examined immunomodulatory effects of quercetin in PBMCs derived from MS patients. We observed a significant effect of quercetin at concentrations of >5 μM. However, this concentration is above the 0.1 μM fasting plasma concentration of quercetin achieved by a normal daily diet containing 23-34 mg of flavonoids [22]. Therefore, this study investigated whether luteolin could exert immunomodulatory effects, at near physiological concentrations when used alone or in combination with interferon beta (IFN-β) upon isolated PBMCs from MS patients.

## Material and methods

### Population

14 relapsing remitting (RR) MS patients (8 females and 6 males) were recruited into the study. Patients were clinically stable with an age ranging between 31-57 yrs (mean 44.9 ± 8.0) diagnosed with MS according to the McDonald criteria [23] and EDSS range between 0-8.0 (mean of 3.6 ± 2.5). Patients were newly diagnosed or were naïve to all disease modifying therapies including IFN-β, glatiramer acetate and natalizumab for the last 6 months, and were not taking glucocorticoids during the last 30 days. Pregnant patients and patients with other inflammatory diseases were excluded from the study. Informed consent, based upon IRB approval protocol was obtained from all subjects.

### Reagents

Luteolin (3',4',5,7-tetrahydroxyflavone), phytohemagglutinin (PHA) and Ficoll-Hypaque were purchased from Sigma Aldrich (St Louis, MO, USA). Luteolin was dissolved in DMSO and added in concentrations that did not exceed 0.05% of the total volume in any of the experiments. IFN-β was obtained from Biogen Idec Inc (Cambridge, MA). RPMI 1640 medium, fetal bovine serum (FBS) and antibiotics were purchased from InVitrogen (Grand Island, NY). Quick Cell Proliferation Assay Kit was purchased from BioVision Inc. (Mountain View, CA). ELISA kits for total MMP-9, and TIMP-1 were purchased from Amersham Biosciences (Piscataway, NJ). ELISA kits for TNF-α and IL-1β were purchased from R&D systems (Minneapolis, MN). Unless otherwise specified all other reagents were of analytical grade.

### Isolation of peripheral blood mononuclear cells

Twenty milliliters of venous blood was obtained from each subject. Peripheral blood mononuclear cells (PBMCs) were isolated using Ficoll-Hypaque isolation technique and cells were washed three times with PBS and counted prior to their use in any experiment. Cell viability was determined by trypan blue dye.

### Proliferation assay

For cell proliferation assays, PBMCs were plated in 96 well tissue culture plates at a density of 5 × 10^4 ^cells/ml, 200 μl/well in complete RPMI 1640 medium. Cells were stimulated with 2 μg/ml of PHA for 48 hrs in the presence of either 0, 0.2, 1, 5,10, 25, 50 μM of luteolin, 2 IU of IFN-β, or a combination of 0. 0.2, 1, 5, 10 μM of luteolin, and 2 IU of IFN-β. PBMCs proliferation was measured by Quick Cell Proliferation Assay Kit..

### Cytokines and chemokines measurements

For measurement of proinflammatory cytokines, MMP-9 and TIMP-1, PBMCs were plated at the density of 1 × 10^6 ^cells/ml in 24-well tissue culture plates in the same culture medium as for cell proliferation assay. Cells were stimulated for 48 h with 2 μg/ml of PHA in the absence or presence of either luteolin and IFN-β, or a combination of luteolin and IFN-β. The concentrations of luteolin and IFN-β were similar to those used for cell proliferation assay. The choice of 2 IU IFN-β is based upon two observations. First, this interferon level is within the steady state concentration of 2-10 IU/ml achieved in patients receiving 6 × 10^6 ^IU of IFN-β (Avonex) given intramuscularly once a week. Second, our own preliminary *in vitro *experiments have shown that 2 IU of IFN-β reduces MMP-9 production by approximately 10-15%. This low percent inhibition was chosen to see possible synergistic effects of IFN-β when combined with various concentrations of luteolin.

Culture supernatants were collected after 48 hours of treatment, spun for 10 min at 2,000 rpm, and stored at -80°C. The proinflammatory cytokines, total MMP-9 and TIMP-1 were all measured by sandwich ELISA as per instructions provided by the manufacturers. The detection limits were 1.9 pg/ml for IL-1β, 15.6 pg/ml for TNF-α and 31.25 pg/ml for both MMP-9 and TIMP-1. All measurements were performed in duplicate, and mean values of the two measurements were used for statistical analysis. Absorbance was measured at 450 nm in a μ Quant Microplate reader system (BioTek Instruments, Inc).

### Statistical analysis

Dose responses of luteolin, for each dependant measure of inflammation (cell proliferation, IL-1β, TNF-α, MMP-9 and TIMP-1) were assessed using analysis of variance with luteolin concentration (0-50 μM) and type of treatment (luteolin single and or combined with 2IU IFN-β). The differences between means were performed using *post hoc *contrasts. Additionally, cell proliferation was included as a covariate in order to control for its effect in models examining luteolin effect on IL-1β, TNF-α, MMP-9 and TIMP-1.

## Results

### Proliferative responses of PBMC's to luteolin

**Figure 1:**
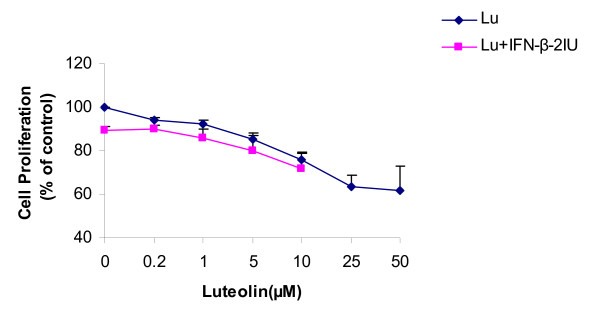
**Dose-dependent effects of luteolin in combination with 2 IU of IFN-β upon PHA-dependent proliferative responses**. PBMCs derived from MS patients were incubated in the presence of 0.2-50 μM luteolin and 0.2-10 μM luteolin in combination with 2IU IFN-β. Values expressed are a percentage of maximum response (% of control). Data is average mean ± SE of 14 MS patients. Abbrev: Lu = luteolin.

Luteolin inhibited PBMCs proliferation in a dose-dependent manner (Figure 1). Luteolin's effect on cell proliferation was significant at concentrations of ≥0.2 μM (reduction of 5.9 ± 1.2% (0.154 ± 0.02 OD vs. 0.145 ± 0.01 OD, *p *= 0.01). At 50 μM luteolin, PBMC proliferation was reduced by 38% with no cytoxicity as indicated by trypan blue dye exclusion test. Similarly, 2IU of IFN-β (Figure 1, luteolin 0 μM) reduced cell proliferation by approximately 8.5 ± 2.1% (*p *< 0.001). IFN-β combined with luteolin reduced cell proliferation by an additional 4.9 ± 2.2% across all luteolin's concentrations, but this additive effect was not statistically significant (*p *values > 0.05).

### Effect of luteolin upon production of pro-inflammatory cytokines by PBMC

**Figure 2:**
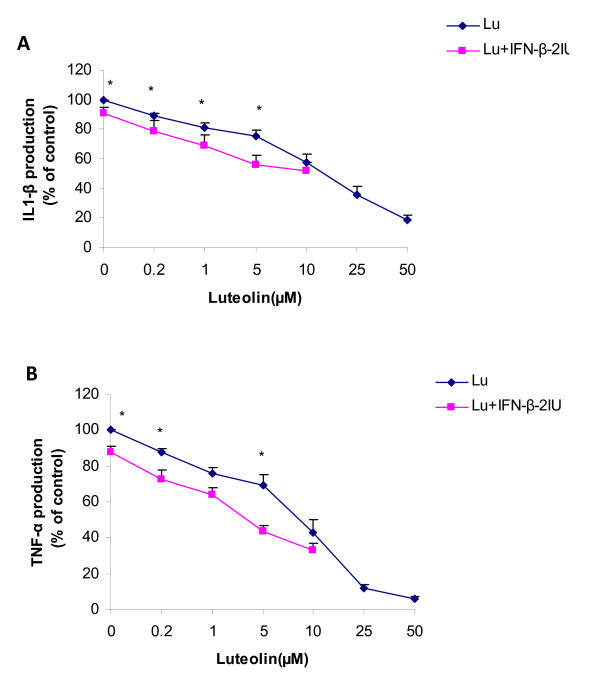
**Dose-dependent effects of luteolin in combination with 2 IU of IFN-β upon PHA-dependent production of IL-1β (2A) and TNF-β (2B) in cell culture supernatant**. PBMCs derived from MS patients were incubated in the presence of 0.2-50 μM luteolin and 0.2-10 μM luteolin in combination with 2IU IFN-β. Values expressed are a percentage of maximum response (% of control). Data is average mean ± SE of 14 MS patients. Asterisks indicate significant differences between luteolin and luteolin in combination with 2IU of IFN-β, P ≤ 0.05 is significant. Abbrev: Lu = luteolin.

Figure 2A and 2B represent the production of IL-1β and TNF-α respectively, in response to luteolin alone and in combination with IFN-β by PBMCs of MS patients. Luteolin reduced the production of IL-1β and TNF-α in a dose-dependent manner. The effect of luteolin was significant at concentrations of ≥0.2 μM with a reduction of 10.7 ± 1.8% (from 882.4 ± 110 pg/ml to 786.5 ± 99 pg/ml, *p *< 0.001) and 12.3 ± 2.2% (from 1988.1 ± 258.3 pg/ml to 1749.8 ± 228.7 pg/ml, *p *< 0.001) in IL-1β and TNF-α respectively. At a concentration of 50 μM, luteolin reduced IL-1β and TNF-α production by 88% and 94% respectively. The effects of luteolin on the production of IL-1β and TNF-α remained significant after correcting for the possible effects of luteolin on cell proliferation (p < 0.001).

IFN-β at 2 IU (Figures 2A and 2B, luteolin 0 μM) reduced IL-1β and TNF-α production by approximately 9.0 ± 4.1% (p = 0.03) and 12.0 ± 3.0% (p < 0.001), respectively. IFN-β combined with luteolin tended to reduce IL-1β and TNF-α production by a respective average of 12.70 ± 2.8% (*p *values between 0.1-0.03) and 15.4 ± 1.5% (*p *values between 0.1-0.02) across all luteolin concentrations.

### Effect of luteolin upon MMP-9 production by PBMC

**Figure 3:**
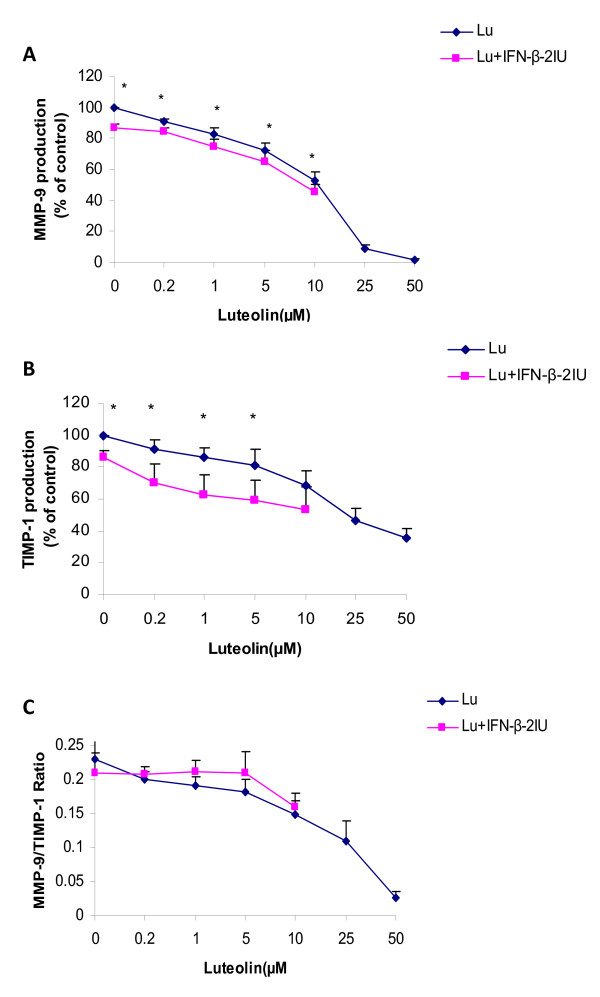
**Dose-dependent effects of luteolin in combination with 2 IU of IFN-β upon PHA-dependent production of MMP-9 (3A) and TIMP-1 (3B) and MMP-9/TIMP-1 ratio (3C) in cell culture supernatant**. PBMCs derived from MS patients were incubated in the presence of 0.2-50 μM luteolin and 0.2-10 μM luteolin in combination with 2IU IFN-β. Values of 3A and 3B expressed are a percentage of maximum response (% of control). Data is average mean ± SE of 14 MS patients. Values of MMP-9/TIMP-1 ratio were obtained by dividing each indicated concentration of MMP-9 with corresponding concentration of TIMP-1. Asterisks indicate significant differences between luteolin and luteolin in combination with 2IU of IFN-β, P < 0.05 is significant. Abbrev: Lu = luteolin.

Luteolin reduced the production of total MMP-9 released by PBMC's from MS patients in the culture supernatant in a dose-dependent manner (Figure 3A). The effects of luteolin on MMP-9 production was significant at concentrations of ≥0.2 μM (reduction of 9.0 ± 1.8%, from 7233.1 ± 1171pg/ml to 6593.8 ± 1095 pg/ml, *p *< 0.001). Luteolin at a concentration of 50 μM reduced MMP-9 production by a maximal of 98%. The effect of luteolin on the reduction of MMP-9 remained significant after correcting for the effect of luteolin on cell proliferation (p < 0.001). IFN-β (2 IU) reduced MMP-9 production by 13.2 ± 0.2% (p < 0.001). 2 IU IFN-β (Figure 3A, luteolin 0 μM) tended to reduce MMP-9 production by an average of 7.3 ± 0.5% (*p *values between 0.02-0.007) across all luteolin concentrations used.

### Effect of luteolin upon TIMP-1 production by PBMC's

Luteolin reduced TIMP-1 production dose-dependently, and this reduction was statistically significance at concentrations of luteolin ≥ 0.2 μM where it reduced TIMP-1 by an average of 8.5 ± 5.9% (from 20748 ± 2515 pg/ml to 19193 ± 2499 pg/ml, p = 0.05). Luteolin at a concentration of 50 μM reduced TIMP-1 by an average of 64%. However, the effect of luteolin on TIMP-1 was not significant after correcting for the possible effects of luteolin on cell proliferation (p = 0.07). IFN-β (2 IU) reduced TIMP-1 production by 13.8 ± 4.2% (p = 0.009) (Figure 3B). The combination of luteolin and IFN-β (Figure 3B, luteolin 0 μM) tended to reduce TIMP-1 by 20.7 ± 5.2% (*p *values between 0.2-0.01) across all luteolin concentrations used.

### MMP-9/TIMP-1 ratio in response to luteolin

Luteolin reduced the ratios of MMP-9 to TIMP-1 released in the culture supernatants. The reduction in MMP-9/TIMP-1 ratio was statistically significant at concentrations of luteolin ≥ 10 μM (from 0.229 ± 0.03 to 0.139 ± 0.02, p = 0.04). IFN-β alone had no effect on this ratio (p = 0.9). MMP-9/TIMP ratio was not different with luteolin compared to luteolin+IFN-β (*p *values between 0.2-0.9) (Figure 3C).

## Discussion

This study provides evidence that luteolin exerts beneficial immunomodulatory effect(s) on PBMCs derived from MS patients. Luteolin, in a dose-dependent manner, reduced PBMC proliferation and production of several pro-inflammatory mediators (IL-1β, TNF-α, and MMP-9) that are crucial in MS pathological processes. The results observed with luteolin are similar to those reported earlier for quercetin [21]. The reduction in production of pro-inflammatory cytokines is independent of the effects of luteolin on cell proliferation. The importance of immune mechanisms in the pathogenesis of MS is well established, since proliferation of myelin-activated cells and the subsequent production of proinflammatory mediators facilitate their migration into the CNS [24], promoting neuronal cell injury.

The effects of luteolin on the degrees of inhibition of IL-1β, TNF-α and MMP-9 were significant at concentrations of 0.2 μM. These inhibitory values are significantly lower than the respective values observed with quercetin in our previous study [21]. The immunomodulatory effects of luteolin at these low concentrations are especially encouraging since these fall in the realm of plasma concentrations of approximately 1.5 μM observed with supplementation of 1 g/day flavonoids [25].

The pharmacological actions of luteolin, combined with its enhanced potency, may be especially attractive in the treatment of MS since, beyond the modulation of peripheral immune cells, luteolin has also been shown to exert immunomodulatory effects inhibiting LPS-induced microglial activation both *in vitro *and *in vivo *[26]. The immunomodulatory effects of luteolin on CNS-resident cells are likely to result in neuroprotection, since there is a linear relationship between extent of microglial activation, demyelination and axonal injury [27]. Similar neuroprotective effects of luteolin have also been observed in rat neural PC12 and glial C6 cells in culture [28,29], and in an *in vivo *model of permanent middle cerebral artery occlusion [29].

Luteolin reduced MMP9/TIMP-1 ratios via a steeper reduction in MMP-9 relative to TIMP-1. This reduction in MMP-9/TIMP-1 ratio further supports a neuroprotective effect for luteolin, since a higher ratio has been shown to be positively associated with disease activity assessed by both the clinical as well as MRI data [30,31]. MMP-9/TIMP-1 ratios are significantly altered in MS lesions [32] and changes in MMP-9 levels are known to contribute to the disruption of the blood-brain barrier and degradation of extra-cellular matrix [33], activities which are regulated by its endogenous inhibitor TIMP-1. Furthermore, MMP-9 can directly cause axonal injury, independent of its effect on blood-brain barrier integrity [34].

**Figure 4:**
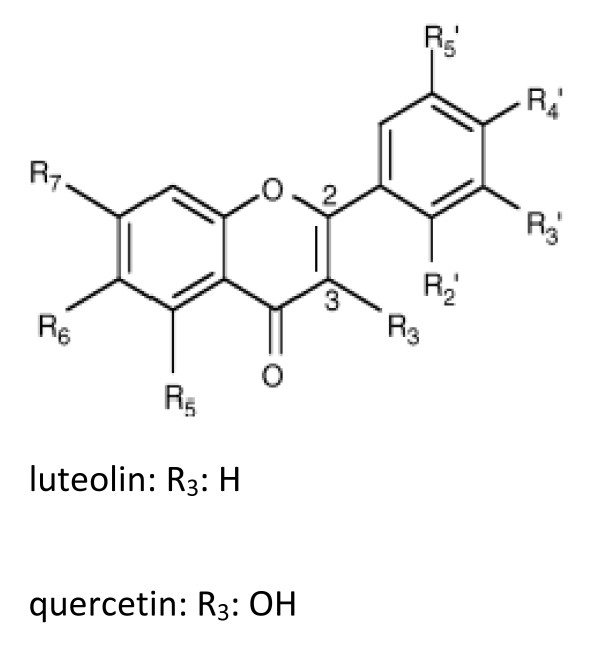
**The structures of quercetin and luteolin**.

Pharmacological effects of flavonoids seem to be dependent on specific structural features of individual flavonoids. Analysis of structure-activity relationships indicate that the immunomodulatory effects of flavonoids depend on the position, number and substitution of the hydroxyl group of the B ring, and on saturation of the C2-C3 bond [18,35]. Although quercetin and luteolin have similar structures (Figure 4), the lack of the hydroxyl group at the C-3 position of luteolin may account for the enhanced immunomodulatory effects observed in this study, as well as luteolin's superior ability to inhibit myelin phagocytosis by macrophages, when compared to quercetin [36].

The immunomodulatory effects of luteolin and related flavonoids has been attributed mainly to the regulation of signaling pathways involving nuclear factor kappaB (NF-kappaB) activation and mitogen-activated protein (MAP) kinase family phosphorylation [10,37]. However, mitogen-induction of NF-kappaB involves many steps including mitogen-induced IkappaB kinase (IKK) activation, IkappaB degradation, DNA binding activity of NF-kappaB complex and its nuclear translocation.

Luteolin has been shown to affect both the upstream (IKK activation (IkappaB degradation) and the downstream (DNA binding activity and nuclear translocation) signaling pathways of LPS-induced NF-kappaB activation in murine macrophages [19,38]. Quercetin, on the other hand, inhibits LPS-induced NF-kappaB activation via inhibiting IKK activation and IkappaB degradation, without affecting DNA binding activity and nuclear translocation of NF-kappaB complex [39,40].

In addition, the treatment of LPS- stimulated RAW 264.7 macrophages with quercetin has been shown to inhibit the activation of phosphorylated ERK MAP kinase and p38 MAP kinase but not JNK MAP kinase [41]. However, luteolin has been shown to inhibit the three MAP kinases in LPS-stimulated macrophages and microglia [42,43]. The differences in the regulation of signaling pathways involved in LPS-induced activation of NF-kappaB and MAP kinases may be related to the structural differences between the two compounds resulting in enhanced immunomodulatory potency of luteolin compared to quercetin.

Nevertheless, this enhanced immunomodulatory effect of luteolin may depend not on the parent compounds, but on their biotransformation to their respective cellular metabolites [44]. The free C-3 hydroxyl group plays an important role in quercetin's immunomodulatory effects [45]. However, depending on the cellular enzymatic pattern, this hydroxyl group is often subject to glucuronidation, glycosylation, methylation or acetylation [46]. The glycosylation or methylation of the hydroxyl group at the C-3 position has been shown to result in the loss of the antiviral activity of quercetin [47]. Similarly, glycolysation at the c-3 position causes loss of quercetin's antiplatelet activity [48], supporting the notion that quercetin metabolites often have reduced immunomodulatory effects compared to the parent compound [49].

The enhanced immunomodulatory effects of luteolin in PBMCs compared to quercetin is consistent with the reported results in the EAE model where luteolin suppressed EAE clinical symptoms more effectively than quercetin [13]. In this latter study, the immunomodulatory effects of luteolin were attributed to the modulation of cytoskeletal regulatory components such as the family of the Rho GTPases. Rho GTPases are known to inhibit NF-kappaB [50]. The inhibition of NF-kappaB could lead to the reduction of MMP-9 [51] observed in our study. It is noteworthy that the efficacy of luteolin in the EAE model is dependent in part on the nature of the antigenic peptide employed, as it has shown to reduce EAE mean clinical scores more effectively in myelin basic protein-induced EAE vs. proteolipid protein-induced EAE [52].

The importance of new compound(s) in the treatment of MS is emphasized by the sub-optimal efficacy of current drugs used in the treatment of MS, including IFN-β, which is the most commonly prescribed disease modifying treatment for relapsing MS. The combination of luteolin and IFN-β enhances the immunomodulatory effects of the latter on most measured variables. Therefore, this combination therapy (luteolin+IFN-β) may improve the clinical efficacy of IFN-β by reducing the level needed for optimal therapeutic effects, hence reducing the likelihood for development of circulating neutralizing antibodies that are known to reduce the efficacy of IFN-β therapy [53].

A review of the data related to the safety of flavonoids support their use as a dietary supplement and as food enrichment [54]. Although, there are no peer reviewed studies on luteolin, the safety of dietary supplementation of 1 g/day of its close relative, quercetin, in humans, has been documented [55]. Moreover, a study in rodents showed no toxicity of luteolin (up to10 mg/kg given IP) administered daily for 18 days, as evidenced by normal food intake and body weight [56].

Nevertheless, low intestinal absorption of flavonoids in general [57] limits their expected beneficial effects in humans. Similar to quercetin, luteolin is found in nature in the form of glycosides [58]. Upon ingestion, luteolin glycosides are cleaved to their biologically active free form (aglycone) in the intestinal mucosa [58]. Luteolin aglycone is subsequently absorbed, and mostly glucuronidated soon after [58]. However, flavonoids' metabolites have reduced biological activities when compared to the parent compound [59].

Human ingestion of bolus dose of 50 mg luteolin has been shown to lead to a peak plasma concentration of 0.05 μmol (total luteolin and its metabolites) after 2 h [60]. This plasma level is similar to the concentration of luteolin aglycone which showed biological activities in our in vitro study. Assuming that a percentage of ingested luteolin could be found in the plasma in the form of aglycone [61], combined with the likelihood of luteolin deglucuronidation during inflammatory processes [62], suggest that luteolin supplementation may lead to its accumulation in tissues [63] such as blood, raising its concentrations to the realm of plasma levels with therapeutic implication in patients with chronic inflammatory and neurodegenerative diseases such as MS.

We have shown that flavonoids luteolin and quercetin are potent *in vitro *inhibitors of proinflammatory markers and could beneficially modulate markers of neuroprotection/neurodegeneration such as the MMP-9/TIMP- ratio. Our *in vitro *observations are consistent with the reported beneficial effects of luteolin when used by MS patients as adjunctive therapy [64], further reiterating the need for ccontrolled, evidence-based clinical trials with flavonoids in general and with luteolin in particular.

## Competing interests

The authors declare that they have no competing interests.

## Authors' contributions

ZS provided the idea, statistical analysis, and manuscript writing. KC provided study supervision and intellectual input. AL provided technical assistance. AD provided research coordination. DH and BW-G recruited patient. FM provided overall study supervision and intellectual input. All authors read and approved the final manuscript.

## References

[B1] Hodek P, Trefil P, Stiborova M (2002). Flavonoids-potent and versatile biologically active compounds interacting with cytochromes P450. Chem Biol Interact.

[B2] Wang LS, Stoner GD (2008). Anthocyanins and their role in cancer prevention. Cancer Lett.

[B3] McCarty MF (2008). Scavenging of peroxynitrite-derived radicals by flavonoids may support endothelial NO synthase activity, contributing to the vascular protection associated with high fruit and vegetable intakes. Medical Hypotheses.

[B4] Mandel S, Weinreb O, Amit T, Youdim MB (2004). Cell signaling pathways in the neuroprotective actions of the green tea polyphenol (-)-epigallocatechin-3-gallate: implications for neurodegenerative diseases. J Neurochem.

[B5] Stadelmann C (2007). Recent advances in the neuropathology of multiple sclerosis. J Neurochem.

[B6] Chen X, Krakauer T, Oppenheim JJ, Howard OM (2004). An injectable multicomponent chinese herbal medicine, is a potent inhibitor of T-cell activation. J Altern Complement Med.

[B7] Verbeek R, Plomp AC, van Tol EA, van Noort JM (2004). The flavones luteolin and apigenin inhibit in vitro antigen-specific proliferation and interferon-gamma production by murine and human autoimmune T cells. J Altern Complement Med.

[B8] Theoharides TC, Kempuraj D, Kourelis T, Manola A (2008). Human mast cells stimulate activated T cells: implications for multiple sclerosis. Ann N Y Acad Sci.

[B9] Kempuraj D, Tagen M, Iliopoulou BP, Clemons A, Vasiadi M, Boucher W, House M, Wolfberg A, Theoharides TC (2008). Luteolin inhibits myelin basic protein-induced human mast cell activation and mast cell-dependent stimulation of Jurkat T cells. Br J Pharmacol.

[B10] Lyu SY, Park WB (2005). Production of cytokine and NO by RAW 264.7 macrophages and PBMC in vitro incubation with flavonoids. Arch Pharm Res.

[B11] Kim JS, Jobin C (2005). The flavonoid luteolin prevents lipopolysaccharide-induced NF-kappaB signaling and gene expression by blocking IkappaB kinase activity in intestinal epithelial cells and bone-marrow derived dendritic cells. Immunology.

[B12] Sharma V, Mishra M, Ghosh S, Tewari R, Basu A, Seth P (2007). Modulation of interleukin-1beta mediated inflammatory response in human astrocytes by flavonoids: implications in neuroprotection. Brain Res Bull.

[B13] Hendriks JJ, Alblas J, Pol SM van der, van Tol EA, Dijkstra CD, de Vries HE (2004). Flavonoids influence monocytic GTPase activity and are protective in experimental allergic encephalitis. J Exp Med Sci.

[B14] Esposito P, Gheorghe D, Kandere K, Pang X, Connolly R, Jacobson S, Theoharides TC (2001). Acute stress increases permeability of the blood-brain-barrier through activation of brain mast cells. J Exp Med Sci.

[B15] Salamon P, Shoham NG, Gavrieli R, Wolach B, Mekori YA (2005). Human mast cells release Interleukin-8 and induce neutrophil chemotaxis on contact with activated T cells. Allergy.

[B16] Ende C, Gebhardt R (2004). Inhibition of matrix metalloproteinase-2 and -9 activities by selected flavonoids. Planta Medica.

[B17] Sellebjerg F, Sorensen TL (2003). Chemokines and matrix metalloproteinase-9 in leukocyte recruitment to the central nervous system. Planta Med.

[B18] Comalada M, Ballester I, Bailon E, Sierra S, Xaus J, Galvez J (2006). Inhibition of pro-inflammatory markers in primary bone marrow-derived mouse macrophages by naturally occurring flavonoids: analysis of the structure-activity relationship. Biochem Pharmacol.

[B19] Xagorari A, Papapetropoulos A, Mauromatis A, Economou M, Fotsis T, Roussos C (2001). Luteolin inhibits an endotoxin-stimulated phosphorylation cascade and proinflammatory cytokine production in macrophages. J Pharmacol Exp Ther.

[B20] Huang YT, Hwang JJ, Lee PP, Ke FC, Huang JH, Huang CJ (1999). Effects of luteolin and quercetin, inhibitors of tyrosine kinase, on cell growth and metastasis-associated properties in A431 cells overexpressing epidermal growth factor receptor. Br J Pharmacol.

[B21] Sternberg Z, Chadha k, Lieberman A, Drake A, Munschauer F (2008). Quercetin and Interferon-beta modulate immune response(s) in Peripheral Blood Mononuclear Cells Isolated from Multiple Sclerosis Patients. J Neuroimmunol.

[B22] Hollman PCH, van Trijp JMP, Mengelers F (1997). Bioavailability of the dietary antioxidant flavonol Quercetin in man. Cancer Lett.

[B23] McDonald WI, Compston A, Edan G (2001). Recommended diagnostic criteria for multiple sclerosis, guidelines from the International Panel on the diagnosis of multiple sclerosis. Ann Neurol.

[B24] Dhib-Jalbut S (2007). Pathogenesis of myelin/oligodendrocyte damage in multiple sclerosis. Neurology.

[B25] Conquer JA, Maiani G, Azzini E (1998). Supplementation with Quercetin markedly increases plasma Quercetin concentration without effect on selected risk factors for heart disease in healthy subjects. J Nutr.

[B26] Jang S, Kelley KW, Johnson RW (2008). Luteolin reduces IL-6 production in microglia by inhibiting JNK phosphorylation and activation of AP-1. Proc Natl Acad Sci USA.

[B27] Noorbakhsh F, Tsutsui S, Vergnolle N, Boven LA, Shariat N, Vodjgani M (2006). Proteinase-activated receptor 2 modulates neuroinflammation in experimental autoimmune encephalomyelitis and multiple sclerosis. J Exp Med Sci.

[B28] Wruck CJ, Claussen M, Fuhrmann G, Romer L, Schulz A, Pufe T (2007). Luteolin protects rat PC12 and C6 cells against MPP+ induced toxicity via an ERK dependent Keap1-Nrf2-ARE pathway. J Neural Transmission.

[B29] Dajas F, Rivera F, Blasina F, Arredondo F, Echeverry C, Lafon L (2003). Cell culture protection and in vivo neuroprotective capacity of flavonoids. Neurotoxicity Res.

[B30] Fainardi E, Castellazzi M, Bellini T (2006). Cerebrospinal fluid and serum levels and intrathecal production of active matrix metalloproteinase-9 (MMP-9) as markers of disease activity in patients with multiple sclerosis. Mult Scler.

[B31] Avolio C, Filippi M, Tortorella C (2005). Serum MMP-9/TIMP-1 and MMP-2/TIMP-2 ratios in multiple sclerosis: relationships with different magnetic resonance imaging measures of disease activity during IFN-beta-1a treatment. Mult Scler.

[B32] Lindberg RL, De Groot CJ, Montagne L (2001). The expression profile of matrix metalloproteinases (MMPs) and their inhibitors (TIMPs) in lesions and normal appearing white matter of multiple sclerosis. Brain.

[B33] Rosenberg GA (2002). Matrix metalloproteinases and neuroinflammation in multiple sclerosis. Neuroscientist.

[B34] Newman TA, Woolley ST, Hughes PM (2001). T-cell- and macrophage-mediated axon damage in the absence of a CNS-specific immune response: involvement of metalloproteinases. Brain.

[B35] Agullo G, Gamet-Payrastre L, Manenti S, Viala C, Remesy C, Chap H (1997). Relationship between flavonoid structure and inhibition of phosphatidylinositol 3-kinase: a comparison with tyrosine kinase and protein kinase C inhibition. Biochem Pharmacol.

[B36] Hendriks JJ, de Vries HE, Pol SM van der, Berg TK van den, van Tol EA, Dijkstra CD (2003). Flavonoids inhibit myelin phagocytosis by macrophages; a structure-activity relationship study. Biochem Pharmacol.

[B37] Kumazawa Y, Kawaguchi K, Takimoto H (2006). Immunomodulating effects of flavonoids on acute and chronic inflammatory responses caused by tumor necrosis factor alpha. Curr Pharm Des.

[B38] Kim JS, Jobin C (2005). The flavonoid luteolin prevents lipopolysaccharide-induced NF-kappaB signalling and gene expression by blocking IkappaB kinase activity in intestinal epithelial cells and bone-marrow derived dendritic cells. Immunology.

[B39] Kim AR, Cho JY, Zou Y, Choi JS, Chung HY (2005). Flavonoids differentially modulate nitric oxide production pathways in lipopolysaccharide-activated RAW264.7 cells. Arch Pharm Res.

[B40] Kim BH, Lee IJ, Lee HY, Han SB, Hong JT, Ahn B (2007). Quercetin 3-O-beta-(2"-galloyl)-glucopyranoside inhibits endotoxin LPS-induced IL-6 expression and NF-kappa B activation in macrophages. Cytokine.

[B41] Cho SY, Park SJ, Kwon MJ, Jeong TS, Bok SH, Choi WY (2003). Quercetin suppresses proinflammatory cytokines production through MAP kinases andNF-kappaB pathway in lipopolysaccharide-stimulated macrophage. Mol Cell Biochem.

[B42] Xagorari A, Roussos C, Papapetropoulos A (2002). Inhibition of LPS-stimulated pathways in macrophages by the flavonoid luteolin. Br J Pharmacol.

[B43] Jang S, Kelley KW, Johnson RW (2008). Luteolin reduces IL-6 production in microglia by inhibiting JNK phosphorylation and activation of AP-1. Proc Natl Acad Sci.

[B44] Kawai Y, Nishikawa T, Shiba Y, Saito S, Murota K, Shibata N (2008). Macrophage as a target of quercetin glucuronides in human atherosclerotic arteries: implication in the anti-atherosclerotic mechanism of dietary flavonoids. J Biol Chem.

[B45] Sin BY, Kim HP (2005). Inhibition of collagenase by naturally-occurring flavonoids. Arch Pharm Res.

[B46] Spencer JP, Kuhnle GG, Williams RJ (2003). Intracellular metabolism and bioactivity of quercetin and its in vivo metabolites. Biochem J.

[B47] Wleklik M, Luczak M, Panasiak W, Kobus M, Lammer-Zarawska E (1988). Structural basis for antiviral activity of flavonoids-naturally occurring compounds. Acta Virol.

[B48] Beretz A, Cazenave JP, Anton R (1982). Inhibition of aggregation and secretion of human platelets by quercetin and other flavonoids: structure-activity relationships. Agents & Actions.

[B49] Lopez-Posadas R, Ballester I, Abadia-Molina AC, Suarez MD, Zarzuelo A, Martinez-Augustin O (2008). Effect of flavonoids on rat splenocytes, a structure-activity relationship study. Biochem Pharmacol.

[B50] Chen LY, Ptasznik A, Pan ZK (2004). RhoA and Rac1 signals in fMLP-induced NF-kappaB activation in human blood monocytes. Biochem Biophys Res.

[B51] Hsieh HL, Yen MH, Jou MJ, Yang CM (2004). Intracellular signalings underlying bradykinin-induced matrix metalloproteinase-9 expression in rat brain astrocyte-1. Cell Signal.

[B52] Verbeek R, van Tol EA, van Noort JM (2005). Oral flavonoids delay recovery from experimental autoimmune encephalomyelitis in SJL mice. Biochem Pharmacol.

[B53] Kappos L, Clanet M, Sandberg-Wollheim M (2005). European Interferon Beta-1a IM Dose-Comparison Study Investigators. Neurology.

[B54] Dunnick JK, Hailey JR (1992). Toxicity and carcinogenicity studies of quercetin, a natural component of foods. Fund Appl Toxicol.

[B55] Conquer JA, Maiani G, Azzini E (1998). Supplementation with Quercetin markedly increases plasma Quercetin concentration without effect on selected risk factors for heart disease in healthy subjects. Fundam Appl Toxicol.

[B56] Fang J, Zhou Q, Shi XL, Jiang BH (2007). Luteolin inhibits insulin-like growth factor 1 receptorsignaling in prostate cancer cells. Carcinogenesis.

[B57] Serra H, Mendes T, Bronze MR, Simplicio AL (2008). Prediction of intestinal absorption and metabolism of pharmacologically active flavones and flavanones. Bioorg Med Chem.

[B58] Shimoi K, Okada H, Furugori M, Goda T, Takase S, Suzuki M, Hara Y, Yamamoto H, Kinae N (1998). Intestinal absorption of luteolin and luteolin 7-O-beta-glucoside in rats and humans. FEBS Lett.

[B59] Lopez-Posadas R, Ballester I, Abadia-Molina AC, Suarez MD, Zarzuelo A, Martinez-Augustin O, Sanchez de Medina F (2008). Effect of flavonoids on rat splenocytes, a structure-activity relationship study. Biochem Pharmacol.

[B60] Shimoi K, Okada H, Kaneko J, Furugori M, Goda X, Takase S, Suzuki M, Hara Y, Kinae N, Kumpulainen JT, Salonen, JT (1999). Bioavailability and antioxidant properties of luteolin. Natural Antioxidants and Anticarcinogens in Nutrition, Health and Disease.

[B61] Shimoi K, Saka N, Kaji K, Nozawa R, Kinae N (2000). Metabolic fate of luteolin and its functional activity at focal site. Biofactors.

[B62] Shimoi K, Saka N, Nozawa R, Sato M, Amano I, Nakayama T, Kinae N (2001). Deglucuronidation of a flavonoid, luteolin monoglucuronide, during inflammation. Drug Metab Dispos.

[B63] Murakami A, Koshimizu K, Ohigashi H, Kuwahara S, Kuki W, Takahashi Y, Hosotani K, Kawahara S, Matsuoka Y (2002). Characteristic rat tissue accumulation of nobiletin, a chemopreventive polymethoxyflavonoid, in comparison with luteolin. Biofactors.

[B64] Namaka M, Crook A, Doupe A, Kler K, Vasconcelos M, Klowak M, Gong Y, Wojewnik-Smith A, Melanson M (2008). Examining the evidence: complementary adjunctive therapies for multiple sclerosis. Neurol Res.

